# Grand challenge in chromatin epigenomics: everything, everywhere, all at once

**DOI:** 10.3389/freae.2023.1195690

**Published:** 2023-05-05

**Authors:** Sharon Y. R. Dent

**Affiliations:** ^1^ Department of Epigenetics and Molecular Carcinogenesis and Center for Cancer Epigenetics, University of Texas MD Anderson Cancer Center, Houston, TX, United States; ^2^ University of Texas MD Anderson Cancer Center/UT Health Houston Graduate School of Biomedical Sciences, Houston, TX, United States

**Keywords:** histone modification, chromatin, development, epigenetics, nucleosome

## Abstract

Our understanding of the regulation and functions of histone modifications has come a long way since they were first reported in the mid-1960s. So too has our understanding of the importance of DNA methylation, histone variants, nucleosome locations and arrangements, and progressively higher order structures that impact when and where DNA-templated processes take place. Recent advances have even allowed the first ever complete sequencing and epigenomic profiles of individual chromosomes from telomere to telomere, including highly repetitive regions that were previously refractory to analysis. The regulatory power of chromatin organization for gene transcription, DNA replication, recombination and repair is undisputable. Still, an ongoing challenge is to understand the full spectrum of changes (everything) that impact processes in cells and tissues (everywhere) and how each change impacts others (all at once).

## Main text

Although a comprehensive review to address such a broad challenge is not feasible in a short article, a few examples focused largely on histone modifications are provided here to illustrate recent advances as well as gaps we still need to fill.

## Everything

In his original report, Vince Allfrey predicted that histone modifications would have regulatory functions (Allfrey, 1966). Hundreds of histone modifications have now been detected by mass spectrometry (Zhao and Garcia, 2015), including acetylation, methylation, phosphorylation, ubiquitylation and many, many others (Figure 1). Individually and in combination, these modifications provide an astronomical number of possibilities for different structural and regulatory states (Zhao et al., 2021; Millan-Zambrano et al., 2022). However, we have only scratched the surface of defining such states and their functions.

**FIGURE 1:**
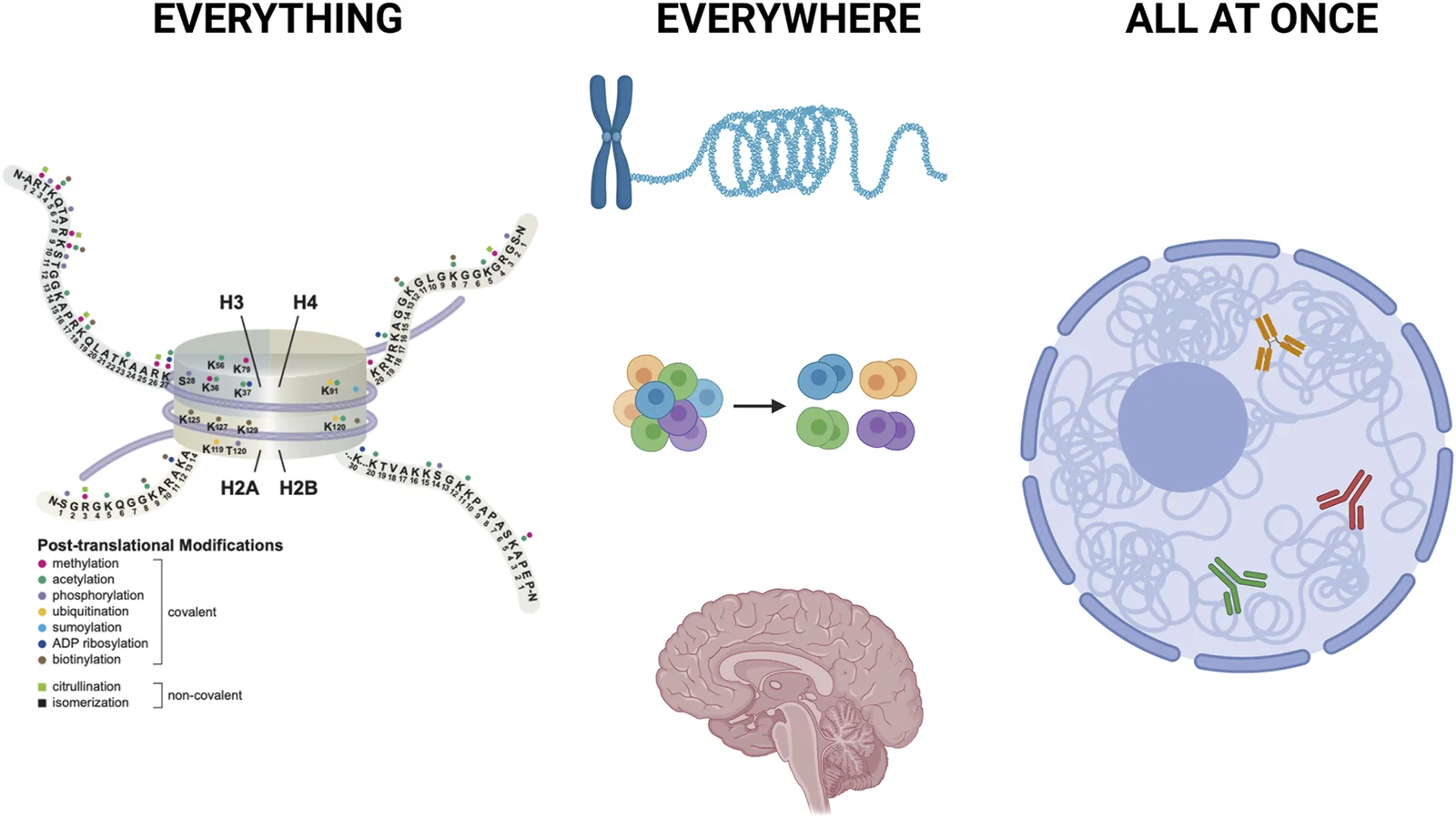
Everything, Everywhere, All at Once Left: A few of the hundreds of histone modifications are shown to illustrate their immense regulatory potential, alone and in combinations. Middle: Recent advances allow definition of histone modification patterns across the genome, including highly repetitive regions such as centromeres and telomeres. In addition, evolving technologies allow analysis of histone modification patterns in single cells, in suspensions and within tissues. Created with BioRender.com. Right: Innovative use of nanobodies allows simultaneous mapping of multiple histone modifications at the same time, in the same nucleus. Combination of those approaches with analysis of chromatin accessibility, active transcription profiles, and cell surface markers provide new insights into cell type specific histone modification patterns that impact gene expression and cell state. Created in part with BioRender.com.

A typical paper these days might report deep data sets defining distributions of select histone modifications, together with DNA methylation profiles and chromatin accessibility measurements associated with changes in gene expression between two or more cellular states. These data sets are powerful, but they run the risk of creating knowledge silos, as we learn more and more about the distributions of only a few histone modification states that correlate well with gene promoter or enhancer activity. For example, our collective database regarding H3K4 monomethylation (me1) or trimethylation (me3) and H3K27me3 genomic distributions is ever growing, while few profiles of other modifications that could be just as important in gene regulation or other processes are generated. To illustrate this point, a search of PubMed reveals almost 3000 papers published between 2012–2022 include H3K4me3 analyses that involve multiple aspects of transcriptional regulation and chromatin organization, in many different biological contexts. In contrast, fewer than 400 papers over this time frame include H2BK120 monoubiquitylation (ub1) analyses, and these mostly report specific proteins involved in the regulation of ubiquitylation and the role of the modification in just a few processes, including gene transcription and DNA recombination. These differences likely reflect the relative lack of reagents to assess occurrence and regulation of H2BK120ub1 relative to H3K4me3, a problem that exists for many post-translational modifications. Overcoming these challenges is critical to truly understanding “everything” about the full regulatory potential of chromatin states.

Given such gaps in our knowledge, a clear distinction between correlations and molecular mechanisms must be maintained. Correlations between gene activity or repression and the presence and absence of specific modifications suggest the modifications may be directly responsible for transcriptional outcomes, but they might actually be secondary to other events (Pollex and Furlong, 2017). For instance, although H3K4me3 is almost universally described as an activating mark due to its presence at gene enhancers and promoters, several studies indicate it may be a consequence of transcription rather than a driver of gene activation (see (Howe et al., 2017) for review). In addition, histone modifying complexes can have non-enzymatic functions that are important for gene regulation (Aubert et al., 2019; Morgan and Shilatifard, 2020).

Genetic approaches provide a powerful avenue for interrogating causality of particular chromatin alterations in gene expression. The importance of specific histone modification sites in gene regulation was first demonstrated by mutation of lysines in histones H4 or H3 in yeast (Kayne et al., 1988; Smith and Stirling, 1988; Megee et al., 1990). Mutation of multiple lysines in the amino terminal tail of H4 to arginine (R) led to loss of silencing at yeast mating type loci, spurring new investigations of the importance of lysine acetylation/deacetylation in heterochromatin formation. In flies, mutation or replacement of H3K27 with arginine (H3K27R) in canonical H3 or variant H3.3 yielded a Polycomb-like phenotype, supporting a critical role for H3K27 methylation in Polycomb-mediated gene silencing during development (Pengelly et al., 2013; McKay et al., 2015). Similar genetic approaches in mammalian cells are hampered by the multiple copies of histone genes and the presence of multiple histone variants. However, gene editing tools are now allowing more direct determination of the “cause vs. effect” of particular modification events to transcriptional outcomes in mouse and human cells. As one example, CRISPR-Cas9 mediated mutation of K27 in both alleles of H3.3 to R in mouse embryonic stem (ES) cells, eliminating both acetylation and methylation of this site, had surprisingly little effect on gene activation or repression (Zhang et al., 2020) However, simultaneous K27R mutations in all 28 alleles of H3.1, H3.2, and H3.3 mimicked the effects of PRC2 deletion in undifferentiated mES cells but had little effect on gene activation during ES cell differentiation (Sankar et al., 2022). These studies indicate that the functions of H3K27 methylation on different H3 variants are redundant but critical for Polycomb-mediated gene silencing, whereas H3K27ac is apparently not required for gene activation, at least in this biological setting.

Histone modification site mutation analyses are complemented by “epigenome editing” approaches wherein specific modifying enzymes or regulatory proteins are directed to particular genomic loci using catalytically inactive versions of the Cas9 protein (dCas9) (Nakamura et al., 2021). dCas9-Ezh2 (O'Geen et al., 2019) and dCas9-p300/CBP (Bohnsack et al., 2017) fusions, for example, have been used to help define the roles of H3K27 methylation and acetylation, respectively, at specific enhancers. Interestingly, directed changes in histone modification states mediated by single factors or enzymes often lead to relatively small and transient changes in gene expression (Nakamura et al., 2021), whereas simultaneous targeting of multiple factors, such as the dCas9-KRAB repressor together with DNA methyltransferases, D-Cas9 DNMT3A or DNMT3A3L, can lead to durable, even heritable, gene silencing (Amabile et al., 2016). Targeting of these same factors to CTCF binding sites displaced CTCF, disrupting promoter-enhancer interactions and higher order TADs (Tarjan et al., 2019).

Altogether, these approaches highlight the fact that no single histone modification is sufficient to fully regulate gene expression. Rather, multiple events work to reinforce each other in a “belts and suspenders” approach to ensure genes are expressed at exactly the right level, at the right time and in the right place, again highlighting the need to assess a greater cross-section of histone modification and chromatin states.

## Everywhere

Of course, deciding where to look for changes in chromatin landscapes is just as important as deciding what is to be examined, both in terms of the genome and in terms of cellular contexts.

On a genomic level, this challenge is exemplified by the lack of analysis of repetitive regions until quite recently. Long-read sequencing methods have now allowed full telomere-to-telomere (T2T) sequence assembly in the pseudo-haploid CHM13 human cell line, filling in gaps and collapsed regions across millions of base pairs generated by limitations in the ability of previous methods to deal with satellite repeats and other highly repetitive regions (Altemose et al., 2022; Gershman et al., 2022; Hoyt et al., 2022). Not only did these studies allow a more complete annotation of previously identified repetitive elements, they identified 43 new repeats and repeat variants, including 19 composite repetitive structures that included genes. Combining these genomic data with nascent RNA-sequencing (PRO-seq) and DNA (CpG) methylation analyses reveals that transcription is low across satellite repeat elements that are hallmarks of centromeres (Gershman et al., 2022). However, abundant transcription of transposable elements provides boundaries for DNA methylation and centromere substructures (Hoyt et al., 2022). The T2T data also provide important new insights to how satellite repeat expansion and decreased DNA methylation shape kinetochore formation and function (Altemose et al., 2022). It will be interesting to compare distributions of histone modifications such as H3K9me3 across these repetitive elements as well. T2T analyses in additional cells and in additional species (everywhere), has great potential to provide new insights gene and genome regulation as well as evolution.

On a tissue level, profiles of histone modification landscapes in total cell suspensions provide an average of changes across different cell types and states, whereas examination of single cells provides a much more refined picture of the range of epigenomic states associated with cell fates and behavior. In the last 2–3 years, combination of highly sensitive mapping techniques such as CUT&RUN (Skene and Henikoff 2017) and CUT&Tag (Kaya-Okur, Wu et al., 2019; Kaya-Okur, Janssens et al., 2020) with single cell analyses has provided unprecedented detail about when and where particular chromatin states matter most. In one example, scCUT&Tag for H3K27me3 together with scATAC-seq to monitor changes in chromatin accessibility enabled identification of cell type-specific Polycomb-silenced loci in solid tissues, in blood, and in tumors (Wu et al., 2021). In another study, comparison of single cell genome distributions of cohesin (Rad21 mapping), a glial cell specific transcription factor (Olig2), and a panel of histone modifications (H3K4me3, H3K27ac, and H3K27me3) identified distinct neural subtypes in oligodendrocyte populations in the mouse brain (Bartosovic et al., 2021). Integration of those mapping data with previously published scRNA-seq (Zeisel et al., 2018) identified even more refined cellular subgroups within these populations. In another example, mapping of HP1alpha, a reader of H3K9me3 which is enriched in heterochromatin, in human colorectal cancer organoids and patient derived xenografts (PDX) together with scATAC-Seq identified changes in heterochromatin compaction associated with tumor drug resistance (Tedesco et al., 2022). These data even allowed measurements of chromatin velocity, defining both the direction of compaction change (opened or closed) as well as the time frame of the changes.

Mapping of chromatin changes “everywhere” must also consider the spatial relationship of cells within tissues. Organs such as the brain, for example, have many types of tissues, made up of multiple types of cells. Cell dispersions that disrupt the connections between cells and between tissues may also alter chromatin patterns and gene expression profiles. One innovative approach to overcome this problem is Spatial CUT&Tag, which generates a two dimensional barcode map linking tissue morphology to histone modification patterns (Deng et al., 2022). This powerful approach allowed definition of changing histone modification patterns during cortical layer development in the mouse brain.

## All at once

To truly define everything, everywhere, we need to be able to monitor multiple events in the same cells, at the same time (all at once). Mapping multiple histone modifications and factors normally requires parallel analyses in separate cells and downstream integration of results. Recent reports, however, describe ways to overcome this limitation by using nanobodies that target different primary antibodies (e.g., mouse or rabbit IgG) (Bartosovic and Castelo-Branco, 2022) or nanobody fusions (Stuart et al., 2022), thereby allowing 2 or 3 different modifications or factors to be mapped simultaneously. Even more dimension can be added by inclusion of scATAC-seq (Bartosovic and Castelo-Branco, 2022) or cell type specific cell surface markers (Stuart et al., 2022; Zhang et al., 2022). Use of nanobodies or barcoded antibodies (Gopalan et al., 2021) that recognize elongating forms of RNA polymerase (pol IIS2P) also allows identification of genes that are actively transcribed. These approaches have proven useful in defining changes in chromatin states during oligodendrocyte differentiation and in identifying immune cell specific patterns of H3K27me3 and H3K27Ac.

Although these innovations are impressive, mapping 2 or 3 modification states at a time still only provides a limited snapshot of the regulatory potential of chromatin given the hundreds of histone modifications that exist. Cytometry Time of Flight (CYTOF) mass spectrometry approaches provide an alternative path towards simultaneous definition of more complex constellations of histone modification patterns. CYTOF combines single cell analysis by flow cytometry with mass spectrometry (Bandura et al., 2009; Iyer et al., 2022) for review). Fluorescent labels are replaced with non-radioactive metal isotopes with unique mass spec signatures. Labeling of antibodies with such isotopes allows quantitative measures of specific proteins or modifications in single cells. Because each tag has a unique signature, use of several antibodies in the same experiment is possible. Cheung et al. developed a panel of heavy isotope labeled antibodies directed against histones and other proteins to enable EpiTOF analyses (Cheung et al., 2018a; Cheung et al., 2018b). This approach allowed simultaneous assessment of levels of eight different histone modifications, total histones, and 4 histone variants along with immune cell specific marker proteins. The data revealed that specific patterns or combinations of histone modifications could predict immune cell identity. Moreover, comparing modification patterns in younger vs. older adults revealed increased variability from person to person and cell-to-cell within an individual with aging. Analysis of 19 pairs of twins indicated that 70% of such changes were not genetically determined, but were likely the result of environmental influences (Cheung et al., 2018a). These findings are reminiscent of studies of DNA methylation changes in twins, which also indicated non-genetic variability with age (McRae et al., 2014; van Dongen et al., 2016).

The power of CYTOF is illustrated by another study that simultaneously profiled levels of 18 different histone modifications, 5 specific histone proteins and 3 regulatory proteins in cells bearing H3.3 K27M mutations (Harpaz et al., 2022). H3.3 K27M is linked to development of a deadly childhood glioma, diffused intrinsic pontine glioma (DIPG) (Wu et al., 2012). Analysis of two patient derived DIPG cell lines that expressed either wild type only or a combination of wild type H3.3 and the H3.3 K27M mutation not only identified differential histone modification patterns between the cell lines, but also identified two populations of cells in the H3.3 K27M expressing line that differed in levels of expression of the mutant histone (Harpaz et al., 2022). Moreover, cells with high expression of H3.3 K27M had elevated levels of several histone modifications associated with active gene transcription (H3, K27Ac, H4K16Ac, H3K4me1/3) and reduced levels of H3S28 phosphorylation and a cleaved form of H3. Cells with lower levels of H3K27M had increased levels of H3K9me3, which is associated with constitutive heterochromatin, and cleaved H3. The presence of H3K27M high and K27M low expressing cells was confirmed in three additional DIPG tumor derived cell lines, and the differential patterns of histone modifications in the two types of cells were highly consistent across the cell lines, and even across the cell cycle. Immunostaining of human tumors also revealed heterogeneity in H3 K27M expression levels. Cell lines expressing high levels of H3K27M were more proliferative and oncogenic than K3K27M low cells. These studies provide new insights to the etiology of DIPGs, and they showcase the full range of impact of changes in expression levels of H3.3 K27M mutation, both direct and indirect, on the histone modification repertoire. The authors also point out the power of comparing cells with a gradient of factor expression levels rather than a binary comparison of cells with and without expression. Their data also suggest a number of co-occurrences in histone modification states, providing fertile ground for additional studies of histone modification cross-talk.

Although CYTOF approaches allow analysis of many, many different chromatin and transcription related parameters “all at once,” they cannot map where in the genome specific changes in histone modifications occur. Perhaps in the future technologies will be able to combine mapping approaches such as CUT&Tag with the unique metal markers used for CYTOF, in a way that does not require obliteration of cells, to add information about genomic locations.

## Final discussion and comments

Chromatin-mediated regulation involves many more features than the few described here and impacts multiple processes beyond transcription. Histone modifications, nucleosome positioning, histone variants, chromatin-associated RNAs, and higher order, 3D chromatin organization influence DNA recombination, replication and repair, mitosis, meiosis, and more. Although this article falls short of truly considering everything chromatin-related, everywhere, all at once, perhaps it will stimulate new conversations and ideas. Finally, this article illustrates how the Chromatin Epigenomics section of Frontiers in Epigenetics and Epigenomics will synergize with the other three sections, especially Epigenomic Tools and Epigenomics and Metabolism.

## Data Availability

The original contributions presented in the study are included in the article/supplementary material, further inquiries can be directed to the corresponding author.
